# Odontogenic Keratocyst Looks Can Be Deceptive, Causing Endodontic Misdiagnosis

**DOI:** 10.1155/2011/159501

**Published:** 2011-10-24

**Authors:** K. M. Veena, Rekha Rao, H. Jagadishchandra, Prasanna Kumar Rao

**Affiliations:** ^1^Department of Oral Medicine and Radiology, Yenepoya Dental College, Yenepoya University, Karnataka, Mangalore 575018, India; ^2^Department of Conservative Dentistry and Endodontics, Century International Institute of Dental Sciences and Research Centre, Poinachi, Kerala, Kasargod 671541, India; ^3^Department of Oral and Maxillofacial Surgery, Yenepoya Dental College, Yenepoya University, Karnataka, Mangalore 575018, India

## Abstract

Odontogenic keratocyst (OKC) is the cyst arising from the cell rests of dental lamina. It can occur anywhere in the jaw, but commonly seen in the posterior part of the mandible. Radiographically, most OKCs are unilocular when presented at the periapex and can be mistaken for radicular or lateral periodontal cyst. When the cyst is multilocular and located at the molar ramus area, it may be confused to ameloblastoma. Lots of cases have been reported in the literature where OKC is associated with the nonvital tooth. So trauma could be one of the reasons in inducing this cyst. In our case, it was in the anterior region at the periapex of nonvital tooth having traumatic occlusion. Hence, the diagnosis of radicular cyst was made and endodontic treatment was done.

## 1. Introduction

Odontogenic Keratocyst (OKC) was first described by Philipsen in 1956. It is the cyst arising from the cell rests of dental lamina. It can occur anywhere in the jaw, but commonly seen in the posterior part of the mandible [1]. It has very aggressive nature and high recurrence rate [2]. The clinical feature and radiographic appearance of OKCs are not characteristic. This may lead to misdiagnosis especially when the lesion is in relation to a nonvital tooth [3, 4]. OKC tends to grow in an anteroposterior direction within the medullary cavity of the bone without causing obvious bone expansion [1]. The radiographic appearance of OKC may range from a small unilocular radiolucency to a large multilocular radiolucency. Hence it may resemble ameloblastoma, dentigerous cyst, lateral periodontal cyst, and radicular cyst [3, 4]. Multiple OKCs are associated with Nevoid basal cell carcinoma syndrome (NBCCS) [2]. Early diagnosis and followup of the patient with OKC is important because possibility of such patient there is a develop to other features of NBCCS in the future.

## 2. Case Report

**Figure 1:**
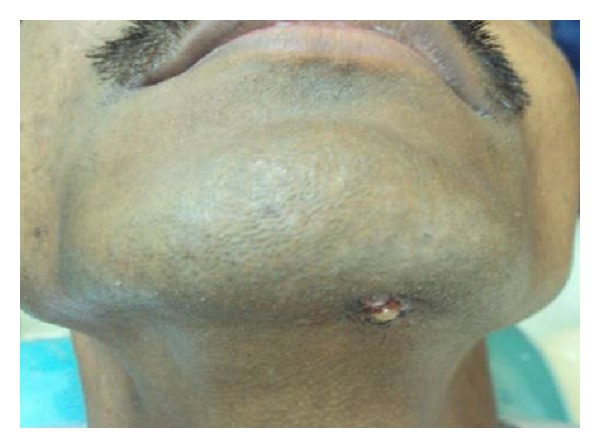
Nonhealing extraoral sinus in the submental region.

**Figure 2:**
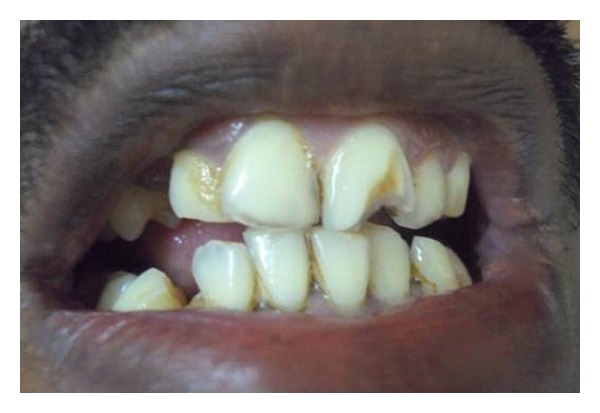
Crowding and traumatic occlusion.

**Figure 3:**
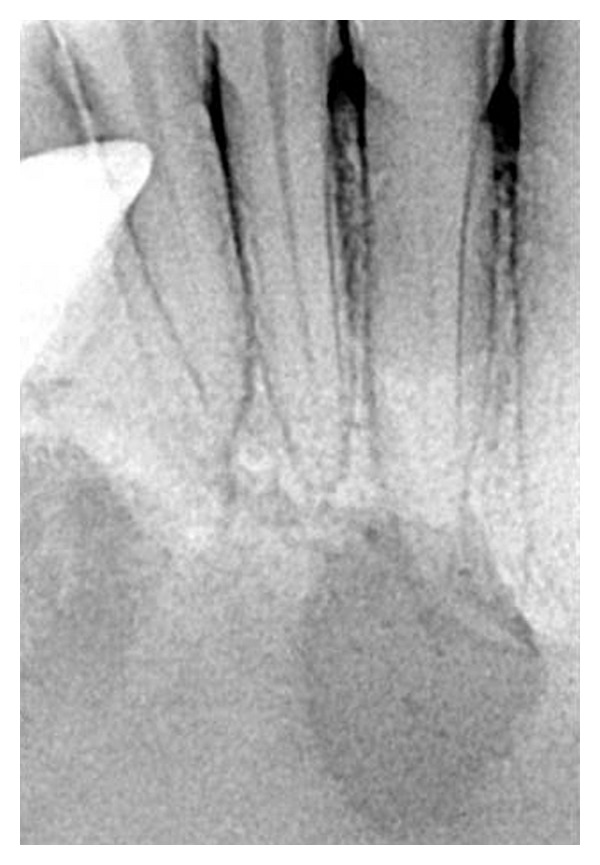
A large, dark radiolucency at the periapex of mandibular left central incisor.

A 25-year-old male patient was referred to a private dental clinic by a dermatologist to rule out any dental cause for a nonhealing extraoral sinus in the submental region (Figure 1). Patient gave a history of intermittent episodes of pain over the past one year. He was on treatment with topical application of various antibiotic ointments prescribed by the medical practitioners. On clinical examination, there was a draining sinus in the submental region, tender on palpation. Intraoral examination showed, crowding of lower anteriors. He had traumatic occlusion causing attrition (Figure 2). Vitality test showed negative response in relation to lower left lateral incisor tooth. Intraoral periapical radiograph was taken which showed a large dark radiolucency at periapex of lower left lateral incisor (Figure 3). Based on history, clinical and radiographic findings, a provisional diagnosis of infected periapical cyst was made. 

It was decided to do the root canal treatment for the nonvital tooth. After complete oral prophylaxis, rubber dam applied. Following access cavity preparation, necrotic tissue was removed, working length determined, and cleaning and shaping of the canal was done. Calcium hydroxide intracanal medicament was given for two appointments in an interval of one week. Later obturation was done with lateral condensation of gutta-percha. But lesion did not show the sign of regression even after three weeks. Hence periapical surgery was decided.

**Figure 4:**
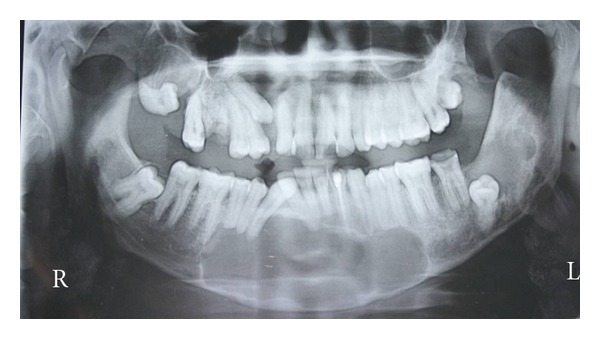
Orthopantomograph shows multiple radiolucencies associated with mandibular anterior region, maxillary right and left as well as mandibular left impacted third molar teeth.

Before the surgery, to know the extent of the lesion, as it could not be made out in intraoral radiograph, an OPG was advised. Surprisingly, orthopantomograph revealed a large radiolucent area in the anterior region of the mandible crossing the midline. It was extending from right premolar region to left premolar area. One more radiolucent area was seen in the left third molar region, which was extending halfway through the ramus, associated with impacted third molar. Cystic radiolucency was seen in the maxillary right and left third molar area also. Here, right third molar tooth was having dilacerations and was horizontally placed along the crest of the alveolar bone. Left maxillary third molar was vertically impacted. Right maxillary canine and right mandibular third molar too were impacted (Figure 4). Since patient had no other features of Nevoid basal cell carcinoma syndrome, diagnosis of nonsyndromic multiple Odontogenic Keratocyst was made. 

**Figure 5:**
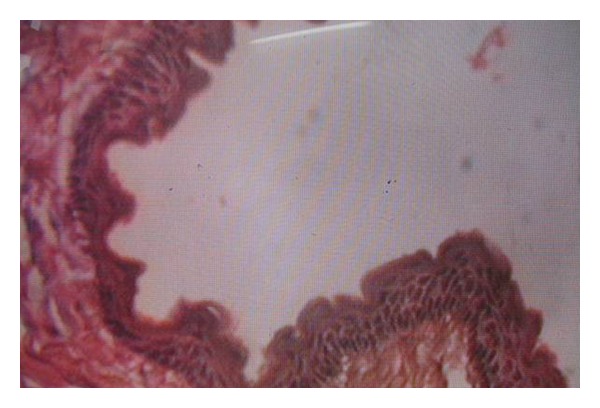
The epithelial lining has 6–8 cell thickness and palisaded basal cell layer.

Patient was referred to an oral and maxillofacial surgeon who performed surgical enucleation of the cystic lesions. Histopathology revealed parakeratotic cystic lining of 6–8 cell thickness with corrugation. The palisading appearance of the basal cells was evident, thus the lesion is confirmed to be OKC (Figure 5). The patient is on regular followup since eighteen months. He has not developed any other cyst or other features of NBCCS till date.

## 3. Discussion

The odontogenic keratocyst is derived from the remnants of the dental lamina with a biologic behaviour similar to a benign neoplasm. Because of this aggressive nature, recently World health organization used the term “keratocystic odontogenic tumor” to describe this cyst [1, 2]. 

It is named keratocyst because the cystic lining produces keratin. The cyst occurs in any age group, but most commonly seen in the second and third decades of life with male predilection. There are no characteristic clinical manifestations. The more common features are pain, soft tissue swelling, expansion of bone, drainage, and paraesthesia of the lip or teeth [2]. Our case was seen in a 25-year-old male patient who had pain and extraoral-nonhealing sinus in the submental region.

Radiographically, most OKCs are unilocular with scalloped margin when presented at the periapex and can be mistaken for radicular or lateral periodontal cyst. When the cyst is multilocular and located at the molar ramus area it may be confused to ameloblastoma. The septa present in ameloblastoma are coarse and curved; originate from the normal bone trapped within the tumor. Hence these septa have honeycomb or soap bubble appearance which is not seen in OKC. In odontogenic myxoma, septa present are thin, sharp, and straight. A simple bone cyst has similar scalloped margin, but this margin is delicate and not distinct [3–5]. In our case, it was in the anterior region at the periapex of nonvital tooth. Hence the diagnosis of radicular cyst was made and endodontic treatment was done.

The odontogenic keratocyst may occur due to traumatic implantation or down growth of the basal cell layer of surface epithelium or reduced enamel epithelium of the dental follicle. Nohl and Gulabivala reported two cases of OKCs, and in their first case, tooth associated with OKC had history of trauma twenty years ago [6]. Our case also had traumatic occlusion. Hence, trauma may be one of the inducing factors in formation of this cyst. 

Multiple OKC occur with some frequency. Many times, it is associated with NBCCS. However, at other times cyst is independent of syndrome. The recurrence rate in these patients is higher may be because, as the number of keratocysts in an individual has, probability of recurrence is higher. As the number of keratocysts increases in an individual, the rate of recurrence also increases [2]. In our case, multiple OKC were found in different regions of the jaws. But other features of the syndrome were not seen.

## 4. Conclusion

OKCs should be one of the differential diagnoses for the periapical radiolucencies which are not responding following the initial endodontic therapy. The clinical, radiographic, and histopathological correlations are essential for proper patient treatment and followup. This will avoid the further complications, since OKCs are highly aggressive, have high recurrence rate, and are associated with NBCCS.
